# Reverse genetics screen identifies six proteins important for malaria development in the mosquito

**DOI:** 10.1111/j.1365-2958.2008.06407.x

**Published:** 2008-10

**Authors:** Andrea Ecker, Ellen S C Bushell, Rita Tewari, Robert E Sinden

**Affiliations:** Division of Cell and Molecular Biology, Imperial College LondonLondon SW7 2AZ, UK

## Abstract

Transmission from the vertebrate host to the mosquito vector represents a major population bottleneck in the malaria life cycle that can successfully be targeted by intervention strategies. However, to date only about 25 parasite proteins expressed during this critical phase have been functionally analysed by gene disruption. We describe the first systematic, larger scale generation and phenotypic analysis of *Plasmodium berghei* knockout (KO) lines, characterizing 20 genes encoding putatively secreted proteins expressed by the ookinete, the parasite stage responsible for invasion of the mosquito midgut. Of 12 KO lines that were generated, six showed significant reductions in parasite numbers during development in the mosquito, resulting in a block in transmission of five KOs. While expression data, time point of essential function and mutant phenotype correlate well in three KOs defective in midgut invasion, in three KOs that fail at sporulation, maternal inheritance of the mutant phenotype suggests that essential function occurs during ookinete formation and thus precedes morphological abnormalities by several days.

## Introduction

Malaria is caused by protozoan parasites of the genus *Plasmodium* that are transmitted between mammalian hosts by female anopheline mosquitoes, in which the parasite completes sexual development. Mosquitoes take up arrested sexual stages (male and female gametocytes) in the blood meal, which are activated by a drop in temperature and the mosquito factor xanthurenic acid. Fertilization usually occurs within 1 h of gametocyte activation and the resulting zygotes differentiate over the next 10–24 h into motile, invasive ookinetes, which exit the blood meal, traverse the midgut epithelium and differentiate beneath the basal lamina into oocysts. In the oocysts repeated rounds of endomitosis result in the formation of thousands of sporozoites, which migrate to the mosquito salivary glands, from where they can be transmitted to the next vertebrate host.

The successful completion of ookinete development and midgut invasion is essential for the establishment of an infection in the mosquito vector, and these processes represent a major population bottleneck in the parasite life cycle. Indeed, while a blood meal can contain thousands of gametocytes, in the field *P. falciparum* oocyst numbers rarely exceed five (Sinden and Billingsley, 2001). Importantly, during this early development in the mosquito the parasite remains extracellular for *c.* 24 h. Antigens expressed during this phase commonly show less polymorphisms than blood stage antigens, as they are not subject to selective pressure by an adaptive immune response (Sinden, 2004). For these reasons, human-to-vector transmission represents an ideal point for intervention, yet the successful application of this concept requires a fuller understanding of parasite development and parasite–mosquito interactions.

To date, only about 25 genes expressed during this crucial phase have been analysed functionally by targeted gene disruption in *P. berghei*, a rodent model parasite often used for the analysis of malaria mosquito stages. In the ookinete, gene targeting studies have largely focused on proteins potentially involved in parasite–host interactions, i.e. surface proteins (e.g. the major ookinete surface proteins Pbs25 and Pbs28) and proteins that are secreted, mainly via specialized secretory apical organelles, the micronemes (e.g. circumsporozoite protein and thrombospondin-related adhesive protein-related protein (CTRP), cell-traversal protein for ookinetes and sporozoites (CelTOS), chitinase, secreted ookinete adhesive protein (SOAP) and members of the LCCL/lectin-adhesive like protein (LAP)/CCp and *Plasmodium* perforin-like protein (PPLP) family (Dessens *et al*., 1999; 2001; 2003; Yuda *et al*., 1999; Tomas *et al*., 2001; Kadota *et al*., 2004; Kariu *et al*., 2006; Ecker *et al*., 2007; Raine *et al*., 2007). Interestingly, ookinete infectivity to the mosquito was completely abolished in only four knockout (KO) lines, *Δctrp, Δpplp3/maop, Δpplp5* and *Δguanylate cyclase (gc)-β*, two of which (*Δctrp*, *Δgc-β*) lack proteins involved in the regulation of ookinete motility or the molecular motor itself. This suggests that significant functional redundancy exists at the ookinete-to-oocyst transition (Dessens *et al*., 1999; Kadota *et al*., 2004; Hirai *et al*., 2006; Ecker *et al*., 2007).

All published reverse genetic studies in *P. berghei* have reported the deletion of no more than three genes. As a consequence of this gene-by-gene approach, a large pool of hypothetical proteins have remained uncharacterized. Indeed, only two genes encoding ookinete proteins without predicted functional domains have been disrupted so far (CelTOS and SOAP) (Dessens *et al*., 2003; Kariu *et al*., 2006). However, transfection methods in *P. berghei* have recently been significantly improved (Janse *et al*., 2006), and together with the availability of the genome sequence of *P. berghei* (Hall *et al*., 2005) and a number of other *Plasmodium* species (Carlton *et al*., 2002; Gardner *et al*., 2002), this finally facilitates a larger-scale approach.

Following the groundbreaking study in *P. falciparum* by Maier *et al*. (2008), who successfully disrupted 53 of 83 attempted genes, elucidating the function of parasite proteins exported to the erythrocyte, this study is the first to describe the larger scale generation and analysis of *P. berghei* mutants, examining 20 proteins that are expressed and putatively secreted by the ookinete. Secreted and surface proteins are not only central to recognition and invasion of target cells, but also to survival within the hostile midgut environment, where parasites are destroyed both by digestive enzymes and immune factors secreted by the mosquito, and by those components of the vertebrate immune system that remain active within the blood meal (Sinden, 2002; Blandin *et al*., 2004; Osta *et al*., 2004). Twelve clonal KO lines were successfully generated and phenotypically characterized in *Anopheles stephensi*. We identify six proteins that are all required during early parasite development in the mosquito, but whose absence results in two distinct mutant phenotypes, depending on when gene function is required. Absence of genes that are expressed *de novo* during ookinete development becomes apparent at ookinete midgut invasion, while lack of genes that must be inherited from the female gametocyte becomes lethal only several days later at sporozoite formation.

## Results

### Selection of candidates

Knockout candidates were selected by a bioinformatic screen of data gathered from a global proteomic analysis of the *P. berghei* life cycle using Multidimensional Protein Identification Technology (MudPIT) (Hall *et al*., 2005), the only comprehensive transcriptional/proteomic data set for the ookinete published to date (Fig. S1). Of 1092 proteins detected in the ookinete proteome, 670 were also detected with high confidence in the asexual blood stages (ABS) and were excluded, thus increasing the chance of selecting proteins involved in processes specific to the ookinete and reducing the risk of lethality of the KO in the (haploid) ABS. As proteins involved in the interactions of the ookinete with the mosquito midgut are likely to be secreted or surface-associated, the remaining 422 proteins were analysed for the presence of a putative signal peptide using SignalP (Nielsen *et al*., 1997). In 74 proteins, a signal peptide was predicted either in the *P. berghei* gene model or in the respective *P. yoelii* or *P. falciparum* orthologues. This subset includes all but one (PPLP5) secreted or surface proteins that had previously been implicated in midgut invasion by targeted gene disruption [CelTOS, chitinase, CTRP, Pbs25, Pbs28, PPLP3 and SOAP (Dessens *et al*., 1999; 2001; 2003; Yuda *et al*., 1999; Tomas *et al*., 2001; Kadota *et al*., 2004; Kariu *et al*., 2006; Ecker *et al*., 2007)], thus validating the selection strategy. From these proteins 20 candidates were selected for targeted gene disruption (Table 1). Proteins were excluded (i) if KOs of their genes have already been reported; (ii) if their gene models, and as a result the SignalP predictions, diverged strongly between different *Plasmodium* species or were classified as ‘uncertain’ on PlasmoDB; (iii) if they had no annotated orthologue in the human parasite *P. falciparum*; and (iv) if their predicted functional domains (e.g. metabolic function) or targeting predictions (apicoplast targeting) made an extracellular role unlikely. If no name had been previously reported in the literature, candidate proteins were named putative secreted ookinete protein (PSOP). Expression data from the *P. berghei* proteome (Hall *et al*., 2005) and information about functional domains predicted for nine of the candidates is given in Table 1. Interestingly, for only three of the candidates [PSOP1, PSOP2 and von Willebrand Factor A domain-related protein (WARP)] were more than 50 spectra collected in the ookinete proteome. In comparison, for five of the eight secreted or surface ookinete proteins previously shown to play a role in midgut invasion were more than 50 spectra recorded. As the number of spectra reflects relative abundance within a proteome (Liu *et al*., 2004), this suggests that the more highly expressed proteins have already been identified using ‘pregenomic’ methods.

**Table 1:**
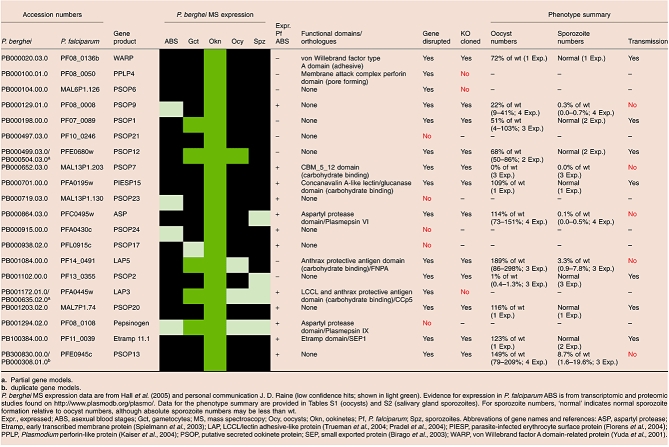
Candidate list and phenotypic summary.

### Generation of clonal KO lines

To functionally characterize the candidate proteins, their encoding genes were disrupted by double homologous recombination, thus replacing the gene of interest with the selectable marker, *T. gondii dihydrofolate reductase/thymidilate synthase (tgdhfr/ts).* Clonal KO lines were obtained by limiting dilution and analysed by pulsed-field gel electrophoresis (PFGE)/Southern blotting and by diagnostic PCR, confirming integration into the correct locus and absence of the wild-type (wt) allele (data not shown), and by RT-PCR on RNA prepared from *in vitro* cultivated ookinetes, confirming absence of the transcript in the respective KO (Fig. S2). Overall, KO clones were successfully generated for 12 candidates (Table 1). This study therefore doubles the number of KO lines available for putatively secreted ookinete proteins. All mutant phenotypes were confirmed in two clones generated in independent transfections, with the single exception of *Δlap5*, for which the phenotype was indistinguishable from KOs of four other members of the *lap* gene family.

For eight candidates at least three independent transfections failed to produce resistant populations from which KO parasites could be cloned (Table 1). For three of these (*lap3*, *pplp4* and *psop6*) integration of the gene targeting construct into the genomic locus was detected in some transfections by diagnostic PCR, suggesting that these genes are not essential in the ABS. However, KOs were never abundant enough in the mixed drug-resistant populations (which also contains episome-containing wt parasites) to allow cloning. It is unclear whether this is due to technical reasons or a reduced growth rate of the KO parasites. For the other five candidates (*pepsinogen*, *psop17*, *psop21*, *psop23*, *psop24*) integration was never observed. We attempted to modify three of these genes (*pepsinogen*, *psop17* and *psop21*) without disrupting gene function by C-terminal c-myc tagging, and succeeded in all cases, confirming that these gene loci can readily be targeted, and thus supporting an essential role of these genes in the ABS (data not shown). While candidates had been selected against detection of protein in the *P. berghei* ABS proteome with high confidence, there is evidence of ABS expression for five of the eight candidates for which no clonal KO lines were obtained (*lap3*, *pepsinogen*, *psop17*, *psop23* and *psop24*) in proteomic (Florens *et al*., 2002; 2004) and microarray studies (Bozdech *et al*., 2003; Le Roch *et al*., 2003) carried out in *P. falciparum*.

### Phenotypic analysis of 12 KO lines in the mosquito host

A phenotypic screen was carried out to identify KO lines with developmental blocks throughout the parasite life cycle in the mosquito host, *A. stephensi* (summarized in Table 1).

From an equal inoculum, all KO lines were able to grow to asexual and sexual parasitaemias similar to wt (data not shown, and Table S1). All KOs produced ookinetes *in vitro*, which appeared morphologically normal in Giemsa-stained culture films (data not shown).

We next determined oocyst numbers, which can be taken as a measure of the ookinete's ability to develop and survive in the blood meal, to recognize and cross the peritrophic matrix, the microvilli-associated network and the midgut epithelial cells and to differentiate into oocysts. At this stage, a striking reduction in infectivity was observed in three KO lines, *Δpsop2, Δpsop7* and *Δpsop9* (Table 1, Table S1). On average, *Δpsop2* produced only 1% of the wt number of oocysts, and *Δpsop9* oocyst numbers were reduced to between 9% and 41% of wt. Most strikingly, *Δpsop7* failed completely to infect most mosquitoes and only two oocysts were observed in a total of 130 mosquitoes. A reduction in oocyst numbers was also observed in *Δpsop1* and *Δpsop12*, but this reduction was weaker, variable and not significant in all experiments. In contrast, *Δasp, Δetramp11.1, Δlap5, Δpiesp15, Δwarp, Δpsop13* and *Δpsop20* all formed oocysts at numbers that were never significantly lower than wt numbers (Table S1).

We then determined salivary gland sporozoite numbers, which illustrate the oocyst's ability to undergo sporulation, and the sporozoite's ability to egress from the oocyst and migrate to the salivary glands. This revealed additional developmental blocks in four KO lines, *Δasp*, *Δlap5*, *Δpsop9* and *Δpsop13* (Table 1, Table S2). In mosquitoes infected with these KO lines only very low numbers of sporozoites were detected in salivary glands preparations and in some experiments *Δasp* and *Δpsop9* sporozoites were completely absent. In all other KO lines, sporozoite numbers mirrored oocyst numbers, i.e. no sporozoites were observed for *Δpsop7*, slightly reduced numbers for *Δpsop1* and *Δpsop2*, and normal numbers for *Δetramp11.1*, *Δpiesp15*, *Δwarp*, *Δpsop12* and *Δpsop20* (Table S2).

Finally, infected mosquitoes were allowed to feed on naïve mice, to establish whether parasites could be transmitted and hence were able to complete their life cycle. The seven KO lines with normal sporozoite production (*Δetramp11.1*, *Δpiesp15*, *Δwarp*, *Δpsop1*, *Δpsop2*, *Δpsop12* and *Δpsop20*) were also infectious to mice, while the five KOs that either failed to produce oocysts (*Δpsop7*) or wt numbers of salivary gland sporozoites (*Δasp*, *Δlap5*, *Δpsop9* and *Δpsop13*) could not be transmitted (Table 1, Table S2).

Based on this phenotypic screen, six KO lines were selected for further study: *Δasp*, *Δlap5*, *Δpsop2*, *Δpsop7, Δpsop9* and *Δpsop13*. Of these, only *Δpsop2* parasites can complete their life cycle, although they suffer a severe population bottleneck before oocyst development. The remaining six KO lines (*Δetramp11.1*, *Δpsop1*, *Δpsop12, Δpsop20, Δpiesp15* and *Δwarp*) were not pursued, because they formed high absolute numbers of oocysts and were easily transmitted through mosquitoes.

### In-depth phenotypic analysis of selected KO lines

#### Defects at ookinete invasion of the mosquito midgut –Δpsop2, Δpsop7 and Δpsop9

A reduction in oocyst numbers may be caused not only by a failure of ookinetes to interact with the mosquito midgut, but also by defects in various other cellular processes at different stages of sexual and sporogonic development. First, to exclude that the reduction or loss of mosquito infectivity of *Δpsop2, Δpsop7* and *Δpsop9* is caused by a failure to form ookinetes *in vivo*, ookinete development was quantified on Giemsa-stained blood films prepared from dissected blood meals. This analysis showed that *Δpsop7* and *Δpsop9* formed ookinetes at numbers that were never significantly lower than wt (Table 2). In contrast, *in vivo* ookinete development of *Δpsop2* was highly variable both between and within experiments (Table 2). For example, in experiments II and III less than 450 *Δpsop2* ookinetes were scored each in nine blood meals, yet the tenth contained 2452 and 7516 ookinetes respectively (versus wt averages of 2433 and 1278 respectively). These data illustrate that while overall ookinete numbers tend to be reduced, in principle *Δpsop2* is capable of producing normal numbers of ookinetes under the variable environments encountered in individual mosquito blood meals. Second, controlling ookinete numbers by feeding *in vitro* cultivated ookinetes at known numbers to mosquitoes via a membrane feeding apparatus did not rescue oocyst formation of *Δpsop2*, *Δpsop7* or *Δpsop9* (Table S3). Taken together, these data demonstrate that the reduced number of oocysts is not secondary to a lack of mature *Δpsop2*, *Δpsop7* or *Δpsop9* ookinetes and must therefore be caused by either impaired ookinete survival, interactions with the mosquito midgut, ookinete-to-oocyst differentiation or early oocyst survival.

**Table 2:** *In vivo* ookinete development of KO clones with reduced oocyst numbers.

Exp.	Parasite	Clone	Gct. (%)	*n*	Mean	SEM	% of wt	*P*-value^a^
I	wt	–	1.4	10	7052	1708	–	–
	*Δpsop2*	1	1.5	10	456	141	6.5	*P* < 0.01
II	wt	–	1.2	10	2433	409	–	–
	*Δpsop2*	1	1.5	10	446	228	18.3	*P* < 0.01
III	wt	–	2.3	10	1278	404	–	–
	*Δpsop2*	2	2.4	10	827	744	64.7	*P* < 0.05
IV	wt	–	2.1	10	1232	669	–	–
	*Δpsop2*	2	1.1	10	8478	1095	688.1	*P* < 0.01
I	wt	–	0.9	10	9779	1778	–	–
	*Δpsop7*	1	1.3	10	6073	1349	62.1	n.s.
II	wt	–	0.4	10	3256	896	–	–
	*Δpsop7*	1	0.6	10	2332	771	71.6	n.s.
III	wt	–	2.0	10	1671	426	–	–
	*Δpsop7*	2	2.3	10	6569	1277	393.1	*P* < 0.01
I	wt	–	2.3	10	1278	404	–	–
	*Δpsop9*	1	2.0	10	2834	661	221.8	n.s.
II	wt	–	2.1	10	1232	669	–	–
	*Δpsop9*	1	2.4	10	1538	486	124.8	n.s.
III	wt	–	1.6	10	2543	384	–	–
	*Δpsop9*	2	1.1	10	2850	694	112.1	n.s.

a Determined by Mann–Whitney *U*-test. Exp., experiment; Gct., gametocytaemia; n.s., not significant.

To differentiate a defect in ookinete invasion from a defect in early oocyst development, which would both ultimately result in a lack of mature oocysts at day 10, ookinete invasion was quantified by feeding *Δpsop2*, *Δpsop7* and *Δpsop9* to *Anopheles gambiae* in which a C-type lectin, CTL4, was silenced by injection of dsRNA (Table 3). CTL4 is a key regulator of melanization of *P. berghei* in *A. gambiae*, and its knock-down (KD) allows the visualization of invading parasites by their melanotic encapsulation as they traverse the midgut barrier (Osta *et al*., 2004). This provides a ‘snap-shot’ of invasion, making visible also those parasites that invade, but later fail to develop into mature oocysts. As shown in Table 3, all KO parasites could successfully be melanized in CTL4 KD mosquitoes. However, less melanized parasites were observed in KO infections compared with wt, suggesting that fewer KO ookinetes invade. Furthermore, the number of melanized KO ookinetes in CTL4 KD mosquitoes was never significantly higher than the number of mature KO oocysts in control LacZ KD mosquitoes, suggesting that those few KO ookinetes that do invade develop successfully.

**Table 3:** Development of KO clones with reduced oocyst numbers in LacZ KD and CTL4 KD mosquitoes.

		dsLacZ (mature oocysts)				dsCTL4 (melanized parasites)				*P*-value^a^	
				
Exp.		Mean	SEM	*n*	Prev. (%)	Mean	SEM	*n*	Prev. (%)	KO versus WT in dsCTL4	dsLacZ versus dsCTL4
I	wt	6	4	10	70	63	25	11	82	–	n.s.
	*Δpsop2*	0.35	0.13	20	30	2.06	0.73	18	39	*P* < 0.01	n.s.
	*Δpsop7*	0.04	0.04	24	4	0.00	0.00	7	0	*P* < 0.01	n.s.
	*Δpsop9*	16	4	40	78	26	7	34	94	n.s.	n.s.
II	wt	11	6	11	45	18	5	18	83	–	n.s.
	*Δpsop2*	0.03	0.03	33	3	0.58	0.29	26	23	*P* < 0.001	n.s.
	*Δpsop7*	0.00	0.00	21	0	0.21	0.07	33	21	*P* < 0.001	n.s.
	*Δpsop9*	4	1	27	59	1	0	28	32	*P* < 0.001	*P*< 0.01

a Determined by Mann–Whitney *U*-test (*n* < 25) or *z*-test (*n* > 25). Exp., experiment; Prev., prevalence; n.s., not significant.

A defect in invasion is further supported by the appearance of midguts 24 h post feed. Midgut epithelia of mosquitoes fed on *Δpsop2-*, *Δpsop7*- and *Δpsop9*-infected mice show only very few extruding epithelial cells, a characteristic sign of invasion (data not shown). To assess the development of *Δpsop7* post midgut invasion, the midgut barrier was bypassed by injecting ookinetes directly into the mosquito haemocoel. This completely rescued mosquito infectivity of *Δpsop7* as assayed by the quantification of salivary gland sporozoites (the quantification of haemocoel oocysts is unreliable) (Table 4). The development of *Δpsop7* oocysts in the haemocoel is thus not affected. The formed sporozoites were also fully infectious to mice by mosquito-bite, but the transmitted parasites remained incapable of infecting mosquitoes. Similarly, *Δpsop2* development post midgut invasion is normal (Table 1). In summary, the lethality of *Δpsop7*, and the significant losses of *Δpsop2* parasites occur at a single point in the parasite life cycle, i.e. at midgut invasion.

**Table 4:** *Δpsop7* salivary gland sporozoite numbers following membrane feeding or haemocoel injection of ookinetes.

			Membrane feeding			Haemocoel injection		
				
Exp.	Parasite	Clone	Mean	*n*	Infectivity to micea	Mean	*n*	Infectivity to mice^a^
I	wt	–	14 730	30	Yes (1/1)	1 596	30	Yes (1/1)
	*Δpsop7*	1	0	21	No (0/1)	3 427	30	Yes (4/4)
II	wt	–	n.d.	n.d.	n.d.	13 399	10	Yes (1/1)
	*Δpsop7*	1	n.d.	n.d.	n.d.	25 323	30	Yes (1/1)
III	wt	–	19 298	30	Yes (1/1)	16 742	30	Yes (1/1)
	*Δpsop7*	2	0	20	No (0/1)	9 837	30	Yes (2/2)

a Number of mice infected/number of mice used. Exp., experiment; n.d., not done.

#### Localization of PSOP2 and PSOP7 by c-myc tagging

**Fig. 1:**
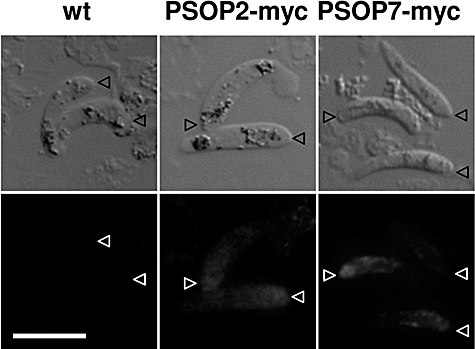
Localization of myc-tagged PSOP2 and PSOP7. Paraformaldehyde-fixed smears of *in vitro* cultivated ookinetes were labelled with α-c-myc rabbit mAb (bottom panels). Top panels show differential interference contrast images. Apical ends of ookinetes are indicated with arrowheads. Scale bar = 10 μm.

C-terminal c-myc tagging of the endogenous gene copies of *psop2* and *psop7* showed that in *in vitro* cultivated, paraformaldehyde-fixed ookinetes both proteins show an apical localization, suggestive of micronemes and thus consistent with a role in invasion (Fig. 1). Apical fluorescence was weaker and more diffuse in *psop2-myc* ookinetes compared with *psop7-myc* ookinetes. Interestingly, PSOP2-myc and PSOP7-myc migrate significantly faster in SDS-PAGE than their predicted molecular weights (Fig. S3), and thus may undergo proteolytic processing. Importantly, both tagged parasite lines show normal mosquito infectivity, indicating that the myc-tagged proteins are functional.

#### Defects in development of oocysts on the mosquito midgut –Δasp, Δlap5, Δpsop13

**Fig. 2:**
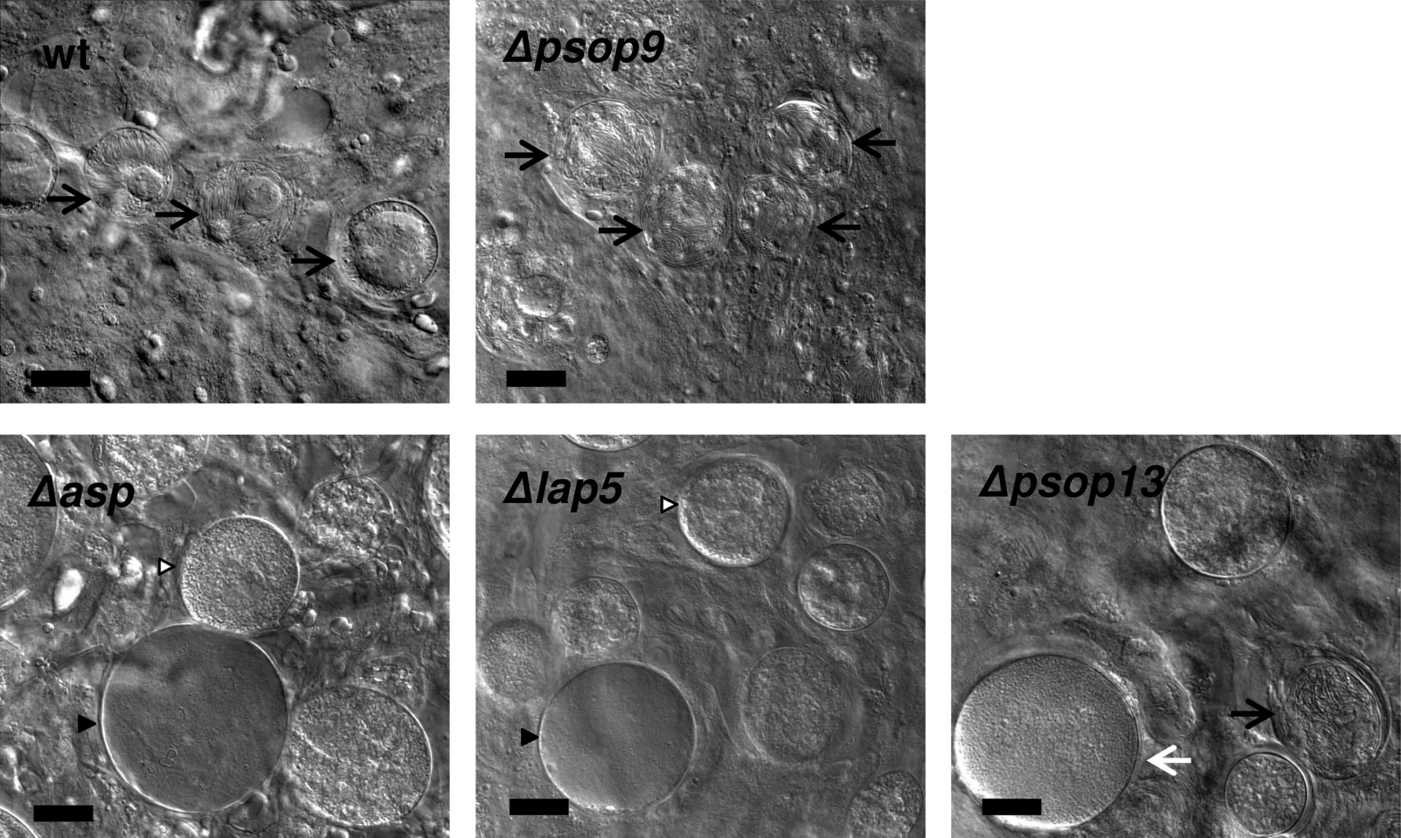
Oocyst morphology of KO clones. Differential interference contrast images of oocysts on days 21–23 of infection in *A. stephensi*. The majority of wt and *Δpsop9* oocysts but only a small proportion of *Δasp*, *Δlap5* and *Δpsop13* oocysts have undergone sporulation (black arrow). Most *Δasp* and *Δlap5* oocysts appear either immature/enlarged (black arrowhead) or degenerate/vacuolated (white arrowhead). Some *Δpsop13* oocysts are enlarged (white arrow). Scale bar = 20 μm.

Three KO lines, *Δasp, Δlap5* and *Δpsop13*, displayed a striking reduction in salivary gland sporozoite numbers despite normal or increased numbers of oocysts (Table 1). This is due to a lack of sporulation and *Δasp*, *Δlap5* and *Δpsop13* oocysts displayed abnormal morphology, ranging from smaller, degenerate to immature enlarged oocysts (Fig. 2). Only in some infections a small number of sporulated *Δasp*, *Δlap5* and particularly *Δpsop13* oocysts were observed and consequently midgut sporozoites were reduced or absent (Table S4). Intriguingly, the mutant phenotype becomes apparent more than 10 days after *asp*, *lap5* and *psop13* are first expressed in the gametocyte/ookinete and is reminiscent of the phenotype reported for *Δlap1* (Claudianos *et al*., 2002), *Δlap2*, *Δlap4* and *Δlap6* (Raine *et al*., 2007).

As both ookinete and oocyst stages are polyploid, a lethal KO phenotype observed at these stages can potentially be complemented by cross-fertilization of KO with wt gametocytes. Indeed, for *Δlap1*, *Δlap2*, *Δlap4* and *Δlap6* we have previously shown that sporulation and transmission can be rescued in heterokaryotic *lap*^-^/*lap*^+^ oocysts provided the functional gene copy is inherited from the female gametocyte. Development was not rescued if only the male gametocyte-derived gene copy was intact, although both male and female gene copies were expressed during sporulation (Raine *et al*., 2007). These results suggest that the essential gene function occurs early during parasite development in the mosquito, when proteomic and reporter studies suggest that only the female gene is expressed (Khan *et al*., 2005). To test whether the same holds true for *Δasp*, *Δlap5* and *Δpsop13*, these KO lines were crossed as previously described (Raine *et al*., 2007) with parasite lines that produce either only functional female (*Δpbs48/45*) or male (*Δpbs47*, *Δnek4*) gametocytes (van Dijk *et al*., 2001; Reininger *et al*., 2005; Mair *et al*., 2006). Here, from similar oocyst infections only crosses with female gametocyte donors were able to establish sporozoite infections of the salivary glands and rescue transmission of the *Δasp*, *Δlap5* and *Δpsop13* parasites (Table 5). Despite this phenotypic difference, RT-PCR analysis of day 10 oocysts derived from these crosses showed that at this time point expression of the male- or female-derived *psop13* gene were similar, while *asp* and *lap5* could not be amplified from cDNA from either crosses (data not shown).

**Table 5:** Sporozoite formation in crosses of sporulation-deficient mutants with male- and female-deficient mutants.

		Oocysts				Sporozoites		
KO clone	Gametocyte donor	Prev. (%)	*n*	Mean	SEM	*n*	Mean	Infectivity to mice^a^
	Male							
*Δasp*	*Δpbs47*	100	20	46	4	20	0	n.d.
*Δasp*	*Δpbs47*	100	10	109	18	30	66	0/2
*Δasp*	*Δpbs47*	100	10	158	29	30	0	n.d.
*Δlap5*	*Δpbs47*	100	10	83	19	31	37	0/1
*Δlap5*	*Δnek4*	100	8	53	29	19	156	0/1
*Δlap5*	*Δnek4*	100	10	68	7	30	59	0/1
*Δpsop13*	*Δpbs47*	100	10	34	9	30	140	0/1
*Δpsop13*	*Δnek4*	90	10	62	10	30	76	n.d.
*Δpsop13*	*Δnek4*	83	10	32	11	30	103	1/1^b^
	Female							
*Δasp*	*Δpbs48/45*	100	20	210	21	20	5 209	n.d.
*Δasp*	*Δpbs48/45*	93	10	101	26	30	28 810	2/2
*Δasp*	*Δpbs48/45*	100	10	54	7	30	13 443	n.d.
*Δlap5*	*Δpbs48/45*	100	10	129	17	18	15 618	1/1
*Δlap5*	*Δpbs48/45*	96	10	89	14	30	3 551	1/1
*Δlap5*	*Δpbs48/45*	100	10	96	15	27	7 258	1/1
*Δpsop13*	*Δpbs48/45*	97	10	25	10	30	13 740	1/1
*Δpsop13*	*Δpbs48/45*	100	10	52	7	30	7 598	n.d.

a Number of mice infected/number of mice used.

b *Δpsop13* allele not transmitted.

Prev., prevalence; n.d., not done.

Importantly, both male and female gametocyte donors were capable of rescuing mosquito infectivity of *Δcdpk3*, *Δctrp, Δpbs25/28, Δpplp5, Δpsop2* and *Δpsop7* (Table S5). These proteins are all required for midgut invasion and these findings demonstrate that they can be provided in time, i.e. within the first 24 h, by the male genome. Should these seven control genes be representative for the male genome overall, we can tentatively narrow down the time point of critical function of ASP, LAP5 and PSOP13 to the same time span, i.e. the first hours of mosquito infection. In summary, the surprising phenotype of an early female-specific defect becoming apparent at the oocyst stage is not unique to the *lap* family, and may represent a more general phenomenon.

#### Defects at the transition of midgut sporozoites to the salivary glands –Δpsop9

In contrast to *Δasp, Δlap5* and *Δpsop13*, *Δpsop9* oocysts are morphologically indistinguishable from wt oocysts (Fig. 2), and produce up to 85% of the wt number of midgut sporozoites (Table S4), which express circumsporozoite (CS) protein on their surface (data not shown). Nevertheless, *Δpsop9* consistently fails to establish a salivary gland infection (Table 1, Table S2). *Δpsop9* sporozoites persist within oocysts at least until day 30 of infection, suggesting that their egress might be impaired. However, *Δpsop9* oocyst sporozoites do not show the abnormal circular arrangement (Fig. 2) and motility that has been reported for another mutant, *Δecp*, that fails to exit oocysts (Aly and Matuschewski, 2005). Like *Δecp, Δpsop9* sporozoites also failed to induce a blood stage infection in mice when 500 000 midgut sporozoites were injected intravenously (0/12 mice infected). In comparison, 100 000 wt midgut sporozoites are sufficient to induce a blood stage infection in mice (Tewari *et al*., 2002) and in our hands, all mice (12/12) became infected when injected with 500 000 wt midgut sporozoites. These data demonstrate that *Δpsop9* sporozoites had also lost infectivity to the vertebrate host or that, alternatively, PSOP9 is required for liver stage development.

## Discussion

The phenotypic characterization of 12 KO lines reported in this study has identified six genes playing key roles in parasite development in the mosquito, as well as six genes that were not critical for mosquito transmission. All candidates were selected based on the possession of putative signal peptides and expression in the ookinete. Despite these shared characteristics lethality was not observed exclusively at the ookinete stage, but at diverse points throughout parasite development in the mosquito, namely at ookinete invasion of the midgut, sporulation and sporozoite egress.

Both PSOP2 and PSOP7 were required at a single point in the parasite life cycle, namely at ookinete invasion of the mosquito midgut. This is consistent with their apical localization in the ookinete and the observation that their transcriptional profile clusters with other genes encoding well-characterized micronemal ookinete invasion-related proteins, such as WARP, SOAP, chitinase and CTRP (E. S. C. Bushell, unpubl. data). Given that sugars have been implicated as mosquito ligands in the binding of ookinetes to the midgut (Zieler *et al*., 1999), it is worth noting that PSOP7 possesses a C-terminal putative carbohydrate binding domain and might thus act as a parasite receptor. Interestingly, only few *Δpsop7* ookinetes were observed attached to the midgut wall 24 h post feed, although they showed normal motility *in vitro* and should therefore be able to exit the blood meal and reach the midgut epithelium (data not shown).

Similar to *Δpsop2* and *Δpsop7*, *Δpsop9* forms reduced numbers of oocysts. In contrast to the former two KOs, which show normal development post midgut invasion, *Δpsop9*, however, suffers an additional developmental block during the transition of sporozoites from oocysts to salivary glands. In line with this dual mutant phenotype, uniquely among the candidates, PSOP9 was detected by mass spectroscopy in all three invasive parasite stages, in *P. berghei* ABS (with low confidence), *P. falciparum* schizonts and merozoites and on the infected RBC membrane, in *P. berghei* ookinetes and in *P. falciparum* sporozoites (Florens *et al*., 2002; 2004; Hall *et al*., 2005; J. D. Raine unpubl. data). However, its function in the ABS is clearly dispensable as KO parasites were readily obtained.

Despite clear evidence for expression during ookinete formation (Hall *et al*., 2005 and Fig. S2), a lethal phenotype due to a lack of ASP, LAP5 and PSOP13 becomes visible only at sporulation. Sporulation represents the end-point of several complex developmental cascades, and would likely be the point where earlier defects in many diverse processes would first become morphologically apparent (Sinden and Matuschewski, 2005). It is thus unclear whether the three individual gene disruptions affect the same pathway/protein complex, or whether the similar morphological changes observed in the absence of seemingly unrelated genes simply are the ultimate result of several independent primary defects. Recently, a role for LAP1 in crystalloid formation was proposed (Carter *et al*., 2008), but the precise cellular functions of the LAPs, ASP and PSOP13 remain enigmatic. Furthermore it is unclear, whether substrates of ASP are of parasite or mosquito origin. The *Eimeria tenella* homologue of ASP, eimepsin, localizes to the refractory body, thereby possibly ensuring equal distribution to daughter cells, but relocalizes to the apical end of sporozoites and merozoites during invasion (Jean *et al*., 2000). Aspartyl proteases of the eimepsin group are suggested to be GPI-anchored, although the GPI-anchor addition sequence is missing from the gene models of the *Plasmodium* orthologues (Shea *et al*., 2007). In *Candida albicans*, GPI-anchored secretory aspartic proteases have been reported to be involved in maintenance of cell surface integrity, cell separation during budding, and host–pathogen interactions, particularly adhesion (Albrecht *et al*., 2006).

Through genetic crosses, we show that, as previously observed in other *Δlap* parasites (Raine *et al*., 2007), the mutant phenotype of *Δasp*, *Δlap5* and *Δpsop13* is maternally inherited. We have previously argued that the failure of male gametocytes to rescue sporulation derives from the absence of expression of the male genome during the very early phase of parasite development in the mosquito, when *de novo* gene transcription might be absent and development might depend on molecules inherited from the female gametocyte as (translationally repressed) RNA or protein (Raine *et al*., 2007). It is therefore worth noting that both the *lap* genes and *asp* show an expression bias towards the female line. In *P. berghei*, LAP1, LAP2 and LAP3 were detected in a female but not a male gametocyte proteome (Khan *et al*., 2005), and in *P. falciparum* expression of LAPs in males appeared weaker than in females and ceased after emergence of microgametes (Scholz *et al*., 2008). *Asp* is one of the best characterized members of a family of genes that are specifically transcribed but translationally repressed in the female gametocyte, are thus thought to function during meiosis in the zygote (Hall *et al*., 2005; Mair *et al*., 2006). Strikingly, the eimepsin transcript was strongly amplified by RT-PCR during meiosis but protein expression lagged behind, similarly suggesting post-transcriptional gene regulation (Jean *et al*., 2001).

Interestingly, in most animal species early development is characterized by a transcriptionally silent phase that is regulated exclusively by maternally inherited components and that lasts until zygotic gene activation, when a major change in gene expression pattern must occur to allow further development (Bouniol *et al*., 1995; Schultz, 1993). It appears that a similar switch from maternal (including ASP, PSOP13 and the LAPs) to zygotic control (including PSOP2 and PSOP7) of development occurs during ookinete formation. While two factors controlling transcription and translational repression in the female gametocyte have recently been identified (Gissot *et al*., 2008; Mair *et al*., 2006), it is still unclear which triggers and factors control the specific *de novo* expression of proteins required in the mature ookinete, such as the invasion-related proteins CTRP, chitinase, PSOP2 and PSOP7.

Our phenotypic screen has also identified several genes whose KOs produced oocysts and salivary gland sporozoites at numbers that were similar to wt (*Δetramp 11.1*, *Δpiesp15*, *Δpsop1, Δpsop12, Δpsop20, Δwarp*). These genes are thus clearly dispensable under the optimal transmission conditions – high gametocytaemias and maximal gametocyte infectivity (Dearsly *et al*., 1990) – of our experimental setting. Nevertheless, we cannot exclude that functional impairments may become apparent under less than ideal circumstances, or in the natural mosquito host of *P. berghei*, *Anopheles dureni*. Alternatively, the failure to observe a mutant phenotype could be due to functional redundancy. Interestingly, α-WARP antibodies can successfully block transmission (Li *et al*., 2004). However, the absence of a functional impairment in *Δwarp* parasites implies that selective pressure by an α-WARP transmission blocking vaccine could potentially lead to loss or significant mutations of WARP without substantial consequences for parasite fitness.

Finally, the five candidate genes that could not be disrupted may encode proteins essential for ABS development, and thus warrant further analysis. Of particular interest is pepsinogen/plasmepsin IX, which in *P. falciparum* is strongly induced during the mid- to late-schizont stage and may thus be involved in merozoite invasion of the RBC (Bozdech *et al*., 2003), explaining our failure to obtain pepsinogen-KO parasites.

## Experimental procedures

### Animals

All experiments involving mice were performed using protocols approved by the British Home Office (Animals Scientific Procedures Act, 1986).

### Generation and molecular characterization of KO and myc-tagged parasite lines

For gene targeting constructs 5′ and 3′ regions of homology of at least 300 bp were amplified from *P. berghei* ANKA clone 2.34 gDNA and successively inserted into pBS-TgDHFR as previously described (Dessens *et al*., 1999). Detailed information on cloning strategies are given in Table S6. All gene targeting constructs are released from the vector backbone using the respective outer restriction sites and integrate into the genomic locus by double homologous recombination. For myc-tagging constructs the 3′ most 1110 bp (pPSOP2-myc) or 1499 bp (pPSOP7-myc) immediately upstream of the stop codon (corresponding to the protein C-terminus) were amplified and cloned into pDR0007 (provided by Dr J. D. Raine) in frame with a tandem double c-myc tag. For transfection, pPSOP7-myc is linearized using a central ClaI site, and pPSOP2-myc using a central EcoRV site, which was introduced by a silent mutation. Myc-tagging constructs were verified by DNA sequencing and integrate into the endogenous locus via single homologous recombination (Fig. S3). All primer sequences are listed in Table S6.

Transfections using the Human T-Cell Nucleofector Kit (amaxa), selection by pyrimethamine and limiting dilution cloning were carried out as previously described (Janse *et al*., 2006). KO clones from independent transfections are labelled 1 and 2 respectively.

Diagnostic PCRs, Southern blotting, PFGE, RT-PCRs and Western blotting were carried out as according to standard procedures or as previously described (Sambrook *et al*., 1989; Janse *et al*., 2006; Raine *et al*., 2007). Antibodies used were α-Pbs28 mouse mAb clone 13.1 and α-c-myc rabbit mAb clone 71D10 (Cell Signalling).

### Phenotypic analysis

General parasite maintenance, ookinete cultures and mosquito infections were carried out as previously described (Sinden *et al*., 2002). Basic phenotyping was carried out in *A. stephensi* SD500. All counting was done blind. Images were taken using a Leica DMR fluorescence microscope and Zeiss AxioCam digital camera.

For immunofluorescence assays (IFAs), ookinete cultures were washed twice in ookinete medium and smeared on microscope slides in a 1:1 mixture of ookinete medium and foetal bovine serum (FBS). Slides were fixed for 10 min in 4% paraformaldehyde in PBS at room temperature, washed once with TBS and permeabilized with 0.2% Triton X-100 in PBS for 5 min at room temperature. IFAs were performed according to the manufacturer's protocol (α-c-myc rabbit mAb clone 71D10; Cell Signalling). All picture processing was identical on all panels. For ookinete injections into the haemocoel an estimated 800 ookinetes in 69 nl of ookinete medium were injected each into the thorax of female adult mosquitoes, using glass capillaries and a microinjector (Nanoject II, Drummond Scientific Company). *In vivo* ookinete development was quantified as described by Alavi *et al*. (2003). Extruding midgut cells were observed by light microscopy on midgut epithelial sheets fixed with paraformaldehyde 24 h post infection as previously described (Ecker *et al*., 2007). For oocyst counts, midguts were dissected into PBS between days 10 and 12 of infection and oocysts counted under phase contrast microscopy. Sporozoite counts were carried out between days 20 and 22 of infection in pools of usually 10 midguts or salivary gland pairs. Midguts or glands were homogenized and sporozoites were quantified in a haemocytometer. The number of sporozoites per mosquito was calculated by dividing total sporozoite counts by the number of mosquitoes with oocysts. Bite-back experiments were carried out between days 18 and 21 of mosquito infection using C57BL/6 mice. Mice were screened for blood stage infections on days 5, 7 and 14 after exposure by examination of tail blood smears. Genetic crosses between different parasite lines were carried out by infecting mice with equal parasite numbers of both clones and allowing mosquitoes to feed directly on these mice. When using *Δpbs47* or *Δpbs48/45*, ookinetes were cultured *in vitro* and fed to mosquitoes at a concentration of 800 ookinetes per microlitre via membrane feeders to reduce leakiness. Diagnostic PCRs on gDNA prepared from either blood, ookinete cultures, infected midguts or transmitted parasites were carried out to confirm that both genotypes were present as described in Raine *et al*. (2007). RNAi KD experiments were performed in *A. gambiae* Yaoundé as previously described (Blandin *et al*., 2002; Osta *et al*., 2004). Briefly, adult mosquitos were injected with dsRNA 1 day post emergence and infected 4 days later. Melanized ookinetes and mature oocysts were counted on day 10 of infection under phase contrast microscopy.
