# Supplementary material for: Reverse genetics screen identifies six proteins important for malaria development in the mosquito
Source: Mol Microbiol. 2008 Oct;70(1):209–20. doi: 10.1111/j.1365-2958.2008.06407.x (PMC2658712; doi:10.1111/j.1365-2958.2008.06407.x)
Supplement: Supplementary file 1 [file mmi0070-0209-SD1.pdf]

Supplementary Figure Legends

Figure S1. Flow diagram outlining the selection of candidate genes

Figure S2. RT-PCR analysis of KO ookinete RNA

RT-PCR analysis on total RNA isolated from purified *in vitro* cultivated KO ookinetes demonstrates absence of transcript in the respective KO. Primers for *pbs28* were used as controls for KO cDNA and wt cDNA was used as positive control for all primer combinations (lower panel). RT, reverse transcriptase; * unspecific

Figure S3. Molecular analysis of myc-tagged parasite lines

A. The gene targeting construct consists of a C-terminal fragment of the gene of interest in frame with a tandem double c-myc tag (M) and a central, unique restriction site (RE). Linearised vector integrates into the targeting locus by single homologous recombination, resulting in the tagging of the endogenous gene copy, and duplication of an incomplete copy. Position of start and stop codons is indicated. Several plasmid copies can integrate in tandem (not shown), producing multiple incomplete gene copies. **B.** Left panel: PFGE analysis of PSOP2-myc (left panel) demonstrates integration into the right genomic locus (chromosome 11). Blot was probed with *pbdhfr 3'UTR* (closed arrowhead, position of chromosome 7 with endogenous *pbdhfr 3' UTR*). Right panel: Southern blot analysis of EcoRV and HindIII digested genomic DNA demonstrates integration of more than one plasmid copy in PSOP7-myc (probed with the C-terminal fragment; expected fragment sizes: wt >7,013 bp; multiple copy integration: 4,378 bp (1st copy) + 9,496 bp (2nd copy) + >15,219 bp (incomplete duplicated copy); *, undigested DNA; open arrowhead, unspecific band). **C.** Western blot analysis of myc-tagged parasite lines. Protein extracts of *in vitro* cultivated, purified ookinetes were separated under denaturing conditions on a 10% SDS-PAGE gel, blotted onto nitrocellulose membranes and probed with a rabbit α-c-myc mAb and reprobed with a mouse α-Pbs28 mAb. Expected molecular weights are 146 kDa for PSOP2-myc and 80 kDa for PSOP7-myc.

Predicted genes in the *P. berghei* genome (Hall *et al.*, 2005): **5,864**

⇩

Proteins detected with high confidence in the *P. berghei* ookinete proteome (Hall *et al.*, 2005): **1,092**

⇩

…of these not detected in the *P. berghei* ABS proteome: **422**

⇩

Signal peptide predicted in either *P. berghei*, *P. yoelii* or *P. falciparum* gene model: **74**

⇩

No KO available: **66**

⇩

Gene models conserved between *Plasmodium* species: **38**

⇩

Genes with orthologues in *P. falciparum*: **33**

⇩

20 candidates

Figure S1

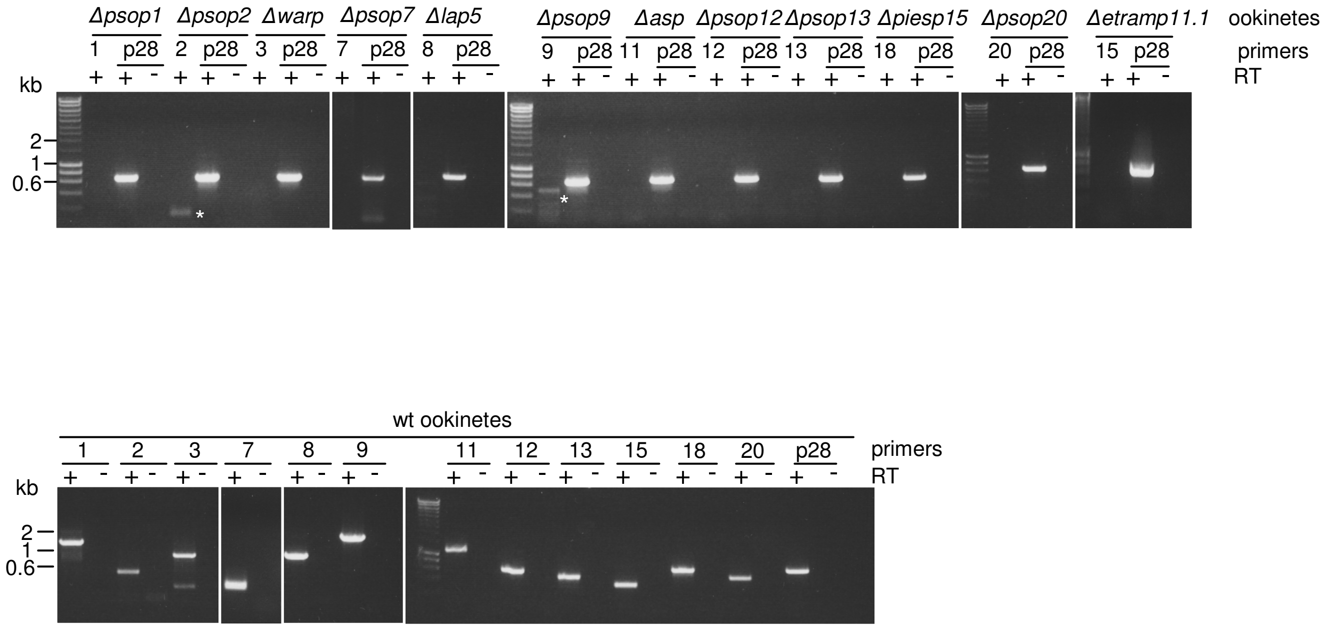
Δpsop1 Δpsop2 Δwarp Δpsop7 Δlap5 Δpsop9 Δasp Δpsop12 Δpsop13 Δpiesp15 Δpsop20 Δetramp11.1 ookinetes
1 p28 2 p28 3 p28 7 p28 8 p28 9 p28 11 p28 12 p28 13 p28 18 p28 20 p28 15 p28 primers
RT
kb
2
1
0.6
*
wt ookinetes
1 2 3 7 8 9 11 12 13 15 18 20 p28 primers
RT
kb
2
1
0.6

Figure S2

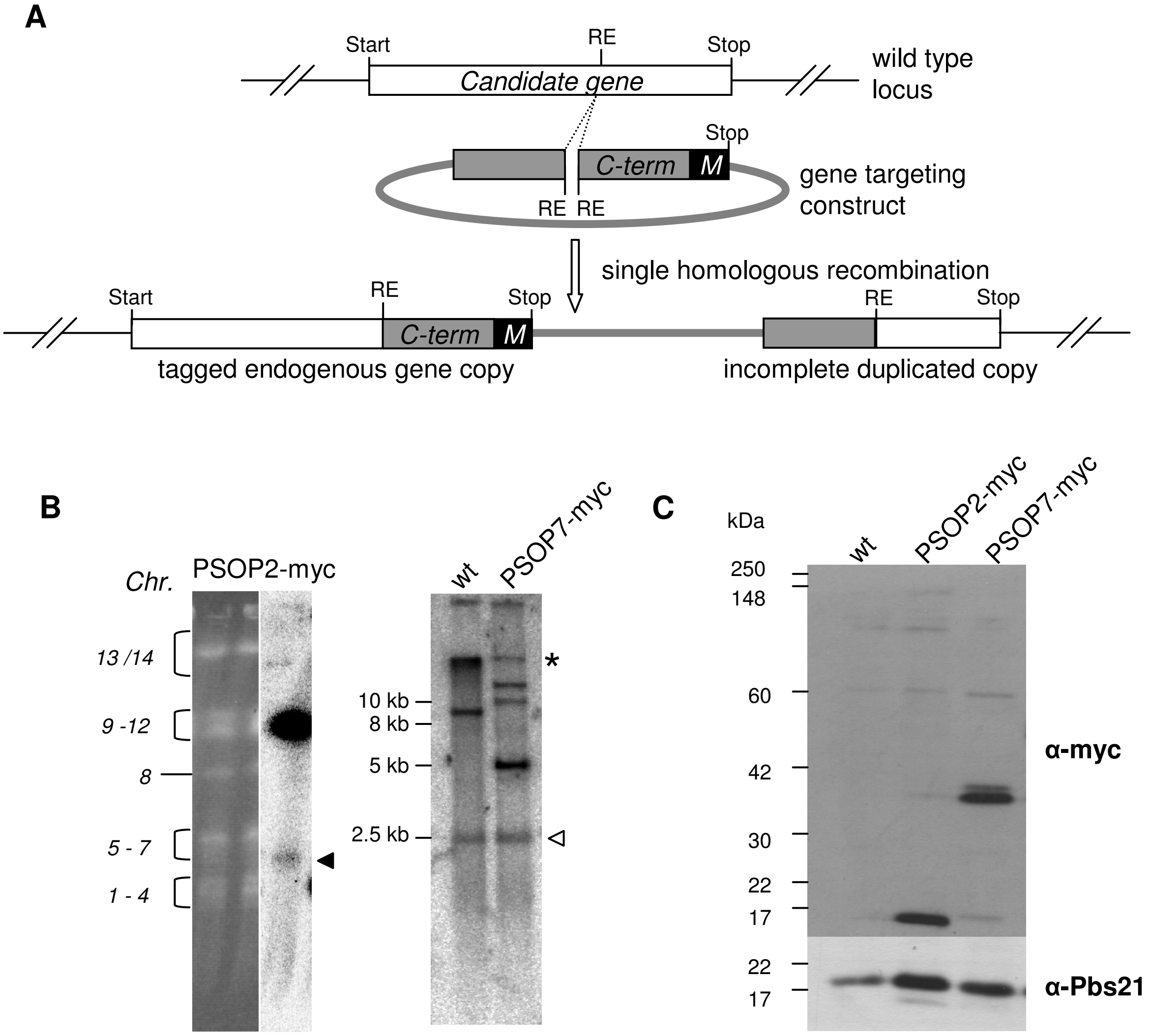
A
Start
RE
Stop
Candidate gene
wild type locus
Stop
C-term
M
RE RE
gene targeting construct
single homologous recombination
Start
RE
Stop
RE
Stop
C-term
M
tagged endogenous gene copy
incomplete duplicated copy
B
Chr.
PSOP2-myc
13 /14
9 -12
8
5 - 7
1 - 4
wt
PSOP7-myc
10 kb
8 kb
5 kb
2.5 kb
*
C
kDa
wt
PSOP2-myc
PSOP7-myc
250
148
60
42
30
22
17
22
17
α-myc
α-Pbs21

Figure S3

Table S1. Oocyst development of KO clones in direct mosquito feeds

KO line	Exp.	Clone	Gct.	n	Prevalence	Mean	SEM	% of wt	P-value[a]
Δasp	A	1	N.D.	50	82%	**224**	27	**72.7%**	n.s.
	B	1	1.6%	50	84%	**241**	24	**109.0%**	n.s.
	C	2	1.3%	35	91%	**113**	18	**150.7%**	n.s.
	D	2	2.1%	50	84%	**66**	14	**122.2%**	n.s.
Δetramp11.1	E	1	2.9%	20	100%	**155**	13	**123.0%**	n.s.
Δlap5	D	1	2.7%	31	94%	**161**	24	**298.1%**	p<0.001
	F	1	3.9%	30	100%	**145**	17	**86.3%**	n.s.
	G	1	1.9%	30	100%	**184**	14	**182.2%**	p<0.001
Δpiesp15	H	1	2.8%	50	86%	**198**	34	**108.8%**	n.s.
Δpsop1	B	1	2.1%	55	89%	**104**	15	**47.1%**	p<0.001
	I	1	N.D.	22	86%	**28**	9	**3.7%**	p<0.001
	J	1	N.D.	50	90%	**39**	6	**102.6%**	n.s.
Δpsop2	A	1	N.D.	50	36%	**1**	0	**0.4%**	p<0.001
	B	1	1.5%	50	52%	**3**	1	**1.3%**	p<0.001
	D	2	1.1%	50	28%	**1**	0	**1.1%**	p<0.001
Δpsop7	K	1	N.D.	50	2%	**0** [b]	0	**0.0%**	p<0.001
	L	1	N.D.	30	0%	**0**	0	**0.0%**	p<0.001
	M	2	2.3%	50	2%	**0** [b]	0	**0.0%**	p<0.001
Δpsop9	D	1	2.4%	50	50%	**6**	2	**11.1%**	p<0.001
	N	1	2.0%	50	74%	**9**	2	**9.0%**	p<0.001
	O	1	2.1%	25	96%	**89**	14	**41.4%**	p<0.001
	P	2	2.5%	50	88%	**38**	5	**28.2%**	p<0.001
Δpsop12	B	1	2.1%	50	88%	**191**	19	**86.4%**	n.s.
	H	1	N.D.	50	76%	**91**	18	**50.0%**	p<0.01
Δpsop13	C	1	0.8%	50	92%	**157**	21	**209.3%**	p<0.001
	D	1	2.6%	50	86%	**105**	15	**194.4%**	p<0.01
	Q	2	2.0%	50	94%	**156**	19	**78.8%**	n.s.
	R	2	1.1%	30	97%	**74**	12	**113.9%**	n.s.
Δpsop20	S	1	2.7%	50	98%	**147**	11	**115.8%**	n.s.
Δwarp	A	1	2.3%	50	78%	**223**	30	**72.4%**	n.s.
**wt**	A	-	2.3%	25	88%	**308**	42	-	-
	B	-	1.2%	52	88%	**221**	22	-	-
	C	-	1.5%	40	75%	**75**	14	-	-
	D	-	2.1%	50	88%	**54**	11	-	-
	E	-	2.2%	20	90%	**126**	17	-	-
	F	-	3.1%	30	93%	**168**	16	-	-
	G	-	2.4%	30	100%	**101**	15	-	-
	H	-	2.1%	50	92%	**182**	23	-	-
	I	-	ND	12	100%	**755**	93	-	-
	J	-	ND	50	78%	**38**	10	-	-

K	-	ND	50	86%	**421**	42	-	-
L	-	ND	13	100%	**317**	36	-	-
M	-	2.0%	30	97%	**145**	22	-	-
N	-	2.3%	35	97%	**100**	18	-	-
O	-	1.9%	27	96%	**215**	20	-	-
P	-	1.7%	50	98%	**135**	14	-	-
Q	-	2.4%	46	98%	**198**	18	-	-
R	-	1.3%	30	93%	**65**	14	-	-
S	-	1.2%	50	98%	**127**	13	-	-

[a] Determined by Mann-Whitney *U* test (n<25) or z-test (n>25); [b] a single oocyst oberved; Gct., gametocytaemia; N.D., not determined; n.s., not significant

Table S2. Salivary gland sporozoite development of KO clones

Parasite	Exp.	Clone	n	Mean	% of wt	Infectivity to mice[a]
Δasp	A	1	29	**0**	**0.0%**	no (0/1)
	B	1	30	**26**	**0.5%**	n.d.
	C	2	10	**0**	**0.0%**	no (0/1)
	D	2	30	**0**	**0.0%**	no (0/1)
Δetramp 11.1	E	1	15	**4,611**	**167.7%**	no (0/1)
	F	1	10	**5,625**	**85.7%**	yes (3/3)
Δlap5	D	1	30	**14**	**0.9%**	no (0/1)
	G	1	28	**55**	**7.8%**	n.d.
	H	1	30	**76**	**1.1%**	n.d.
Δpiesp15	I	1	30	**5,019**	**87.2%**	yes (1/1)
Δpsop1	B	1	30	**3,224**	**66.7%**	n.d.
	J	1	36	**1,854**	**14.8%**	yes (1/1)
Δpsop2	A	1	30	**181**	**4.7%** ‡	yes (1/1)
	B	1	30	**1,146**	**23.7%**	n.d.
	D	2	30	**826**	**53.4%**	yes (1/1)
Δpsop7	K	1	30	**0**	**0.0%**	no (0/1)
	L	2	29	**0**	**0.0%**	no (0/1)
	M	2	30	**0**	**0.0%**	n.d.
Δpsop9	D	1	30	**0**	**0.0%**	no (0/1)
	N	1	10	**38**	**0.7%**	n.d.
	O	1	30	**8**	**0.3%**	no (0/1)
	P	2	30	**63**	**0.3%**	no (0/1)
Δpsop12	B	1	29	**7,679**	**158.8%**	n.d.
	I	1	30	**5,600**	**97.3%**	yes (1/1)
Δpsop13	C	1	15	**93**	**19.6%**	no (0/1)
	D	1	30	**25**	**1.6%**	no (0/1)
	Q	2	30	**263**	**4.9%**	n.d.
Δpsop20	R	1	30	**2,757**	**67.2%**	yes (1/1)
Δwarp	A	1	30	**11,900**	**306.7%** [b]	yes (1/1)
**wt**	A	-	3	**3,880**	-	n.d.
	B	-	30	**4,836**	-	n.d.
	C	-	17	**475**	-	yes (1/1)
	D	-	30	**1,547**	-	yes (1/1)
	E	-	10	**2,750**	-	no (0/1)
	F	-	10	**6,563**	-	yes (2/2)
	G	-	30	**708**	-	n.d.

H	-	20	**7,070**	-	n.d.
I	-	38	**5,755**	-	yes (1/1)
J	-	30	**12,519**	-	n.d.
K	-	26	**6,018**	-	n.d.
L	-	30	**4,252**	-	n.d.
M	-	10	**5,160**	-	n.d.
N	-	10	**5,700**	-	n.d.
O	-	30	**2,784**	-	yes (1/1)
P	-	29	**20,638**	-	yes (1/1)
Q	-	20	**5,410**	-	n.d.
R	-	30	**4,105**	-	yes (1/1)

[a] Number of mice infected/number of mice used; [b] estimate as n (of wt)=3 ; n.d., not done

Table S3. Oocyst development of KO clones in ookinete membrane feeds

Parasite	Exp.	Clone	n	Prevalence	Mean	SEM	% of wt	P-value[a]
Δpsop2	A	1	50	60%	**1.52**	0.26	**0.50%**	p<0.001
	B	2	50	26%	**0.32**	0.08	**0.21%**	p<0.001
	C	2	45	24%	**0.31**	0.09	**0.21%**	p<0.001
Δpsop7	D	1	30	0%	**0.00**	0.00	**0.00%**	p<0.001
	D	1	50	0%	**0.00**	0.00	**0.00%**	p<0.001
	E	2	50	2%	**0.02**	0.02	**0.01%**	p<0.001
Δpsop9	B	1	33	58%	**0.82**	0.15	**0.54%**	p<0.001
	C	1	50	70%	**2.40**	0.48	**1.64%**	p<0.001
	F	2	28	86%	**6.07**	1.05	**20.23%**	p<0.001
**wt**	A	-	50	100%	**302**	19	-	-
	B	-	23	100%	**153**	12	-	-
	C	-	38	100%	**146**	9	-	-
	D	-	30	100%	**152**	14	-	-
	E	-	30	93%	**137**	13	-	-
	F	-	34	91%	**30**	5	-	-

[a] Determined by Mann-Whitney U test (n<25) or z-test (n>25)

Table S4. Midgut sporozoite development of KO clones

Parasite	Exp.[a]	Clone	n	Mean	% of wt
Δasp	C	2	10	**0**	**0.0%**
	D	2	30	**0**	**0.0%**
	S	2	20	**11,784**	**7.6%**
Δlap5	D	1	30	**418**	**5.2%**
	G	1	28	**5,915**	**16.6%**
	H	1	30	**9,689**	**18.3%**
Δpsop9	D	1	30	**4,462**	**55.7%**
	N	1	10	**4,950**	**12.9%**
	O	1	30	**11,248**	**84.6%**
	P	2	30	**21,801**	**27.2%**
Δpsop13	C	1	15	**1,271**	**53.4%**
	D	1	30	**1,638**	**20.4%**
	Q	2	30	**30,856**	**46.0%**
**wt**	C	-	17	**2,378**	-
	D	-	30	**8,014**	-
	G	-	30	**35,652**	-
	H	-	20	**52,885**	-
	N	-	10	**38,353**	-
	O	-	30	**13,291**	-
	P	-	29	**80,162**	-
	Q	-	20	**67,104**	-
	S	-	10	**155,125**	-

[a] Same experiment names as in Table S2

Table S5. Oocyst formation in crosses of midgut invasion-deficient mutants with male- and female-deficient mutants

KO clone	Gametocyte donor	Oocysts Prevalence	n	Mean	SEM
	**MALE**				
Δcdpk3	*Δpbs47*	100%	24	**77**	9
Δcdpk3	*Δpbs47*	89%	37	**3**	0
Δcdpk3	*Δpbs47*	100%	35	**139**	11
Δctrp	*Δpbs47*	98%	40	**97**	9
Δctrp	*Δpbs47*	100%	34	**72**	6
Δpbs25/28	*Δpbs47*	98%	20	**20**	2
Δpbs25/28	*Δpbs47*	100%	32	**25**	3
Δpplp5	*Δpbs47*	96%	26	**20**	4
Δpplp5	*Δpbs47*	96%	49	**33**	3
Δpsop2	*Δpbs47*	96%	25	**8**	1
Δpsop2	*Δpbs47*	100%	50	**56**	3
Δpsop2	*Δpbs47*	100%	9	**55**	5
Δpsop7	*Δpbs47*	97%	37	**18**	2
Δpsop7	*Δpbs47*	100%	25	**72**	8
	**FEMALE**				
Δcdpk3	*Δpbs48/45*	100%	30	**71**	8
Δcdpk3	*Δpbs48/45*	100%	15	**147**	13
Δctrp	*Δpbs48/45*	100%	40	**89**	7
Δctrp	*Δpbs48/45*	38%	21	**1**	0
Δctrp	*Δpbs48/45*	100%	48	**60**	4
Δpbs25/28	*Δpbs48/45*	100%	44	**132**	8
Δpbs25/28	*Δpbs48/45*	98%	41	**42**	3
Δpplp5	*Δpbs48/45*	92%	26	**16**	5
Δpplp5	*Δpbs48/45*	96%	26	**76**	8
Δpsop2	*Δpbs48/45*	100%	40	**12**	1
Δpsop2	*Δpbs48/45*	98%	50	**134**	7
Δpsop2	*Δpbs48/45*	100%	15	**33**	4
Δpsop7	*Δpbs48/45*	100%	20	**7**	1
Δpsop7	*Δpbs48/45*	95%	20	**67**	8

Table S6. Primer sequences and cloning strategies

A. Generation of gene targeting constructs

Candidate	Homology region	Primer combination:<br>Primer name (**restriction site**), primer sequence (5'→ 3')	Size (digested)	Strategy
asp	5'	AE11a (ApaI), TT**GGGCCC**TGTAATTTGTACTTGCACACGAG<br>AE11b (HindIII), CC**AAGCTT**TTGGTTATATATAGACAATGTTG	526 bp	gene disruption (fragment encoding C-terminal 27 amino acids retained)
	3'	AE11c (EcoRI), T**GAATTC**TAGAAAATATTACACAATATTTG<br>AE11d (BamHI), GG**GGATCC**GCTACAATAAATGGAATAAGTAC	288 bp	
etramp 11.1	5'	AE15a (ApaI), TT**GGGCCC**TTTATATTTTCTTATAGTGCAGG<br>AE15b (HindIII), CC**AAGCTT**GGGAATTAATGTAATTATAAGAG	595 bp	entire coding region removed
	3'	AE15c (EcoRI), T**GAATTC**ATCTAGCACAAAACCAGTACCAG<br>AE15d (BamHI), GG**GGATCC**ACACACATCACGGCACTATTTAC	546 bp	
lap3	5'	AE04 a (ApaI), TT**GGGCCC**TAAGAGATTGAGTGAGGCTATTC<br>AE04b (HindIII), CC**AAGCTT**ACGACTTGTTTAAAATAGAATGG	569 bp	entire coding region removed
	3'	AE04c (EcoRI), T**GAATTC**GATTAAAATTTTAGTTCGGTTTC<br>AE04d (BamHI), GG**GGATCC**TGGAAATGCAAAAAATAAAGATG	487 bp	
lap5	5'	AE08a (ApaI), TT**GGGCCC**TGTTATATATTGCACATATAGCC<br>AE08b (HindIII), CC**AAGCTT**GGTATAACGTCAAGTTATAATTG	562 bp	entire coding region removed
	3'	AE08c (EcoRI), T**GAATTC**CAAACTGACAAATGAAATATGCC<br>AE08d (BamHI), GG**GGATCC**ATTTTGTGCAATCCCAGATGTAC	427 bp	
pepsinogen	5'	AE14a (KpnI), GG**GGTACC**TTGTCTGTTATACATATACCCTC<br>AE14b (endogenous HindIII site), TTGGGCCCTCAGTTTTTATTAACGTTGTTAC	365 bp	entire coding region removed
	3'	AE14c (EcoRI), ATGGGAGAG**GAATTC**ATAAATAG<br>AE14d (BamHI), GG**GGATCC**AGTTTTAAATTGCATACGAAAAC	444 bp	

piesp15	5’	AE18a (KpnI), GG**GGTACC**ACTGCCGTTTTGCACATATTGAC<br>AE18b (ApaI), TT**GGGCCC**ACACTATGGAGCTTTCACAGGTG	566 bp	gene disruption (fragment encoding C-term. 119[Py] amino acids retained)
	3’	AE18c (EcoRI), T**GAATTC**ATATTCATAATATGCAAGATGAG<br>AE18d (Sac II), TCC**CCGCGG**AATATGTTTTTTTTCAACATCTC	356 bp	
pplp4	5’	AE10a (ApaI), TT**GGGCCC**GATAATATATACATAGGTGTTTG<br>AE10b (HindIII), CC**AAGCTT**CGTTATCGATACAATTTGTATTC	545 bp	gene disruption (fragment encoding C-terminal 133 amino acids retained
	3’	AE10c (EcoRI), T**GAATTC**GTTCTATAGGTGATAATAAGTGT<br>AE10d (BamHI), GG**GGATCC**ATTTCACCATAACAGTAGATAAC	468 bp	
psop1	5’	AE01a (ApaI), TT**GGGCCC**ACATATACATGTATGTACACCTG<br>AE01b (HindIII), CC**AAGCTT**CTGTCTTATTAAGATTCGAGATG	464 bp	entire coding region removed
	3’	AE01c (EcoRI), T**GAATTC**TTGCACAGGATGACATAGATGGC<br>AE01d (BamHI), GG**GGATCC**CATACCTACTCCCATGTGTGCAC	486 bp	
psop2	5’	AE02a (ApaI), TT**GGGCCC**TAATTGATTCTGAACATGTCAGG<br>AE02b (HindIII), CC**AAGCTT**GAAAAAACCAAAAGTTAAAGCAC	466 bp	entire coding region removed
	3’	AE02 c (EcoRI), T**GAATTC**TATATGTATGCGTTCACATATTG<br>AE02d (BamHI), GG**GGATCC**AGCATGTTGACATGTATATTATG	589 bp	
psop6	5’	AE06a (ApaI), TT**GGGCCC**GGATCTTTGTAACATTTGGCATG<br>AE06b (HindIII), CC**AAGCTT**TTATATCCACACAGTGTAAGAG	461 bp	entire coding region removed
	3’	AE06c (EcoRI), T**GAATTC**AGTAAAATGCACACACAACATTC<br>AE06d (BamHI), GG**GGATCC**TTGGCATAACATATAATTTCACG	584 bp	
psop7	5’	AE07a (ApaI), TT**GGGCCC**AATGTTCACATGAACATATAAGG<br>AE07b (HindIII), CC**AAGCTT**CGTATATGTATAGGTGGTGCTAA	562 bp	entire coding region removed
	3’	AE07c (EcoRI), T**GAATTC**TAGCATATACATAATAATGCTGC<br>AE07d (BamHI), GG**GGATCC**TGAGTATAAAAACTCATCTTTCC	558 bp	

psop9	5’	AE09a (ApaI), TT**GGGCCC**ATTTTATATACCCGTAATTAATG<br>AE09b (HindIII), CC**AAGCTT**TAACATATAAATATATATACACC	309 bp	entire coding region removed
	3’	AE09c (EcoRI), T**GAATTC**GTATCATGCTATTTATTACATAC<br>AE09d (BamHI), GG**GGATCC**AGGAAATACAAATTAGAATAACC	538 bp	
psop12	5’	AE12a (ApaI), TT**GGGCCC**GTCATTATCAGCTTGTAAATAGG<br>AE12b (HindIII), TAA**AAGCTT**TCAATTTATGTGTC	487 bp	entire coding region removed
	3’	AE12c (EcoRI), T**GAATTC**GTTTCTTATTGCACACACATACA<br>AE12d (BamHI), GG**GGATCC**ATCCGCTAAAGATGCATTGAATG	585 bp	
psop13	5’	AE13a (ApaI), TT**GGGCCC**AATAACTATCGTTTTTACAACG<br>AE13b (HindIII), CC**AAGCTT**TGATGCGATACTAAATATTACG	542 bp	entire coding region removed
	3’	AE13c (EcoRI), T**GAATTC**GTAAGATAAATATTTTGTTGCCT<br>AE13d (BamHI), GG**GGATCC**TTTTATACCATTCCTGAAAGATG	543 bp	
psop17	5’	AE17a (ApaI), TT**GGGCCC**CAAAATTACCATTCTAATGCAAC<br>AE17b (HindIII), CC**AAGCTT**CAGAATTTGAGCAATAACACTAG	625 bp	entire coding region removed
	3’	AE17c (EcoRI), T**GAATTC**TCCATCAAAAATGTTTGCTTAAC<br>AE17d (BamHI), GG**GGATCC**ATAAACGTGTGCATTTATGTGTG	605 bp	
psop20	5’	AE20a (ApaI), TT**GGGCCC**TAAGGCATGAATAAGAATGATAC<br>AE20b (HindIII), CC**AAGCTT**GTAATAATAGGTTTTTAACCATC	467 bp	gene disruption (fragment encoding C-terminal 29 amino acids retained)
	3’	AE20c (EcoRI), T**GAATTC**GTAAAAATACACACATACGCATG<br>AE20d (BamHI), GG**GGATCC**TAATTAGTTTTCATCATATGGAG	333 bp	
psop21	5’	AE21a (ApaI), TT**GGGCCC**CAAAGGGAATATGGAATATTTAG<br>AE21b (HindIII), CC**AAGCTT**ACATCATCAGTATTTTTACAATC	315 bp	gene disruption (fragment encoding N-terminal 502^{Pf} amino acids retained)
	3’	AE21c (EcoRI), T**GAATTC**ATATTAATAAGTTAGGCGAAACC<br>AE21d (BamHI), GG**GGATCC**TGCACATATTATATGCTTAACTG	565 bp	

psop23	5’	AE23a (ApaI), TT**GGGCCC**ATTAGCAATATGGTGTGTACATG<br>AE23b (HindIII), CC**AAGCTT**CAAGAATCAATAATTTAAAAGTAG	543 bp	entire coding region removed
	3’	AE23c (EcoRI), T**GAATTC**AAATTGTCTAAAAGAGGGAATGC<br>AE23d (BamHI), GG**GGATCC**CACACATAAATATGTGCATTATC	535 bp	
psop24	5’	AE24a (KpnI), GG**GGTACC**CAAGGGGGAAAG*T*TGAAAATTG<br>AE24b (HindIII), TT**GGGCCC**CTTTTCCGCTCTCATTCCTTATG	362 bp	gene disruption (fragments encoding N- term. 116 and C-term. 456[Pf] amino acids retained)
	3’	AE24c (EcoRI), T**GAATTC**ACAGCATATTTTAGAAAATACTG<br>AE24d (BamHI), GG**GGATCC**TTAATATAATTTTCATCATATTC	336 bp	
warp	5’	AE03a (KpnI), GG**GGTACC**TTCCTGATTGTTCATACGGAAAG<br>AE03b (endogenous HindIII site), TTGGGCCCTATTTTTGTGGGTGAGGAAAGC	384 bp	entire coding region removed
	3’	AE03c (EcoRI), T**GAATTC**AATGCCTTATTAGAAACCGAAAC<br>AE03d (BamHI), GG**GGATCC**CAAGCGCATTTAACCATTTTAAC	525 bp	

[Py] or [Pf], size of retained fragment based on *P. yoelii* or *P. falciparum* gene models, respectively

B. Generation of myc-tagging constructs

**Candidate**	**Primer combination:**<br>Primer name (**restriction site**), primer sequence (5’→ 3’)	**Size**
psop2	#2 c-myc a (KpnI), GG**GGTACC**GAAAATGCCATAGATCGAGTTG<br>#2 c-myc b (EcoRV), CGT**GATATC**TTTTGCATATTTTTCAC<br>#2 c-myc c (EcoRV), GTGAAAAATATGCAAAA**GATATC**ACG<br>#2 c-myc d (ApaI), TT**GGGCCC**TAATTTTTTATTTTTCTTTTCAACTG	1,110 bp
psop7	#7 c-myc F (KpnI), GG**GGTACC**CGAATGCCATAAGTGATAATCAG<br>#7 c-myc R (ApaI), TT**GGGCCC**AAAATTGTAACAATCACTTGTTTTGTC	1,499 bp

C. Diagnostic PCRs

**Candidate**	**Allele**	**Primer combination**[a]:<br>Primer name, primer sequence (5'→ 3')	**Size**
asp	WT	11KO, ATTAGCGGAAGTTGAAAAGGAAC<br>11WT, CAGTGGATATTCTTATCGATG	761 bp
	KO	11KO<br>248, GATGTGTTATGTGATTAATTCATACAC	854 bp
etramp 11.1	WT	AE15a (see A.)<br>AE15d (see A.)	1,540 bp
	KO	15KO, GAGACATTTATGAATTAAATGTTTTC<br>248	992 bp
lap3	WT	4KO, TCCGACTTATTGAAAGAGCACAG<br>4WT, GCAAAGCCTTCCATCCAATG	935 bp
	KO	4KO<br>248	1,007 bp
lap5	WT	8KO, CACATTGATGTTGGGAATAAACTC<br>8WT, GTCATAATTCTCACTGTAGC	896 bp
	KO	8KO<br>248	950 bp
pepsinogen	WT	AE14a (see A.)<br>AE14d (see A.)	2,774 bp
	KO	14KO, GTTGTCGAATTTGTTATATAGGAC<br>248	943 bp

piesp15	WT	18KO, GGTGAAATAACAAATTAGAACAAAGTG<br>18WT, ATTTGTTGACTCCCCCTCAC	985 bp
	KO	18KO<br>248	958 bp
pplp4	WT	10KO, TGTTCATGCAATTTGCAGCTTAC<br>10WT, TTGGTAATGGCCACCCAAAC	911 bp
	KO	10KO<br>248	924 bp
psop1	WT	1KO, TCACGTGTTAATAAGTATTTGTC<br>1WT, CACCATCGATATCTGTGTAC	843 bp
	KO	1KO<br>248	832 bp
psop2	WT	2KO, GTGTGCACACTATGATATAACTC<br>2WT, TCTACAACTATGGCTCTCTC	917 bp
	KO	2KO<br>248	825 bp
psop6	WT	6KO, AAAGGACCTAAAGAAAATAACCA<br>6WT, GCGACCAGCCTATATAAATG	943 bp
	KO	6KO<br>248	853 bp
psop7	WT	7KO, CTCATGATACACAAAGAAATAAGC<br>7WT, TTTCAGCATATGCACTTCGC	758 bp
	KO	7KO<br>248	840 bp

psop9	WT	9KO, TTTATTTTGTTATCGTTTTTTTTAATA<br>9WT, TGCTTACTTCAGGGACTAAG	530 bp
	KO	9KO<br>248	621 bp
psop12	WT	12KO, CACATTGCAAAGTCAGGCCATTA<br>12WT, ATCATTACACACTTGGACTATTTG	821 bp
	KO	12KO<br>248	890 bp
psop13	WT	13KO, CAATACTAATATTGTGGTGATGATC<br>13WT, TTCGGTGAAACAGCATCTGC	870 bp
	KO	13KO<br>248	935 bp
psop17	WT	AE17a (see A.)<br>AE17d (see A.)	1,840 bp
	KO	17KO, CCATTGATTTCTCATATCCGTAAC<br>248	1,097 bp
psop20	WT	20KO, GATCATAATATAGCGCATACTTTC<br>20WT, GCACAAATATCTTCTATAAGTGC	711 bp
	KO	20KO<br>248	791 bp
psop21	WT	AE21a (see A.)<br>AE21d (see A.)	4,050 bp
	KO	21KO, CAAAAAATAATAAAATAGTTAATGCAG<br>248	575 bp

psop23	WT	AE23a (see A.)<br>AE23d (see A.)	2,337 bp
	KO	23KO, GTTCATATAAATTATTCCCAGAC<br>248	936 bp
psop24	WT	AE24a (see A.)<br>AE24d (see A.)	1,656 bp
	KO	24KO, AAACGATCAAATTTAAAATACATAAAG<br>248	674 bp
warp	WT	3KO, ACGATAGTAGACAGCTAAAATAC<br>3WT, TCATCAATGCCCAAATCTCC	817 bp
	KO	3KO<br>248	831 bp

[a] forward primer "KO" binds upstream the 5' homology region; reverse primer "WT" binds in the sequence that is removed from the genome

D. RT-PCR

**Candidate**	**Primer combination:**<br>Primer name, primer sequence (5'→ 3'; gene-specific sequence underlined)	**Size**
asp	#11 expr F1, GACGACGACAAGATGAAAAATACCTTTTCAATGAAATAAAAT<br>#11 expr R , GAGGAGAAGCCCGGTTTAAAAATTGTGATTGCTTTTAACTAC	1,214 bp
etramp 11.1	15F, GCGAGATCTATGAAACTAGCAAAAGCATTATATTTT<br>15R, GCGCCTAGTGATGGTGGTACACTGTCAT	558 bp
lap5	LAP5-2 F, GACGACGACAAGATGGAATATAAAAAATTAGGAGGTGTATATG<br>LAP5-2 R, GAGGAGAAGCCCGGTTCAACTGCGTATAGCTTTCGATTTTTC	941 bp

pbs28	p28F, GCGAGATCTATGAATTTTAAATACAGTTTTATTTTTTTA<br>p28R, GCGCCTAGCATTACTATCACGTAAATAACAAGTA	642 bp
piesp15	18RT-F, TTAGATTATTCAGCAGCTTCCAC<br>18RT-R, GGGTAAGAGAAACTGACTTATC	664 bp
psop1	1F, GCGAGATCTATGAATAAACTTTTAATGCTATCTC<br>1R, GCGCCTAGGCTTACTCGACACAAACATCG	1,440 bp
psop2	#2 N-term F, GACGACGACAAGATGAGTATTGAAAATAAAAATAATATAAAC<br>#2 N-term R, GAGGAGAAGCCCGGTTTATATATCACAATTTCTCAGTTTATCAG	465 bp
psop7	#7seqF, CTCATTTGATGATAAAGTTGAAACAG<br>#7seqR, ATCATTTCCATTGTCACTTTGTG	301 bp
psop9	9F, GCGAGATCTATGAAACGCAATATATTATTCCCTT<br>9R, GCGCCTAGAATGAAATATAAAAAAATAGAAAGCAAAG	1,875 bp
psop12	AE19c, TGAATTCCACCTTCTTCGTAACAGGAGTTG<br>AE19d, GGGGATCCCATGTTTAGCACCATTATAAACC	590 bp
psop13	13RT-F, GCGCTGTTGCCTTTTTCG<br>13RT-R, ATAGTGTATTACCTTCTTCGGG	393 bp
psop20	20RT-F, GGTTATAATGAATGCTTATGTG<br>AE20d (see A.)	495 bp
warp	3F, GCGAGATCTATGAAGAGTGTTAAAGGAATAACATA<br>3R, GCGCCTAGATTTTTTATCGTCGTTTTTCTTTCAG	912 bp
